# Telemedicine Cognitive Behavioral Therapy for Anxiety After Stroke: Proof-of-Concept Randomized Controlled Trial

**DOI:** 10.1161/STROKEAHA.120.029042

**Published:** 2020-07-24

**Authors:** Ho-Yan Yvonne Chun, Alan J. Carson, Athanasios Tsanas, Martin S. Dennis, Gillian E. Mead, Clementina Calabria, William N. Whiteley

**Affiliations:** 1 Centre for Clinical Brain Sciences (H.Y.Y.C., A.J.C., M.S.D., G.E.M., W.N.W.), University of Edinburgh, UK.; 2 Centre for Medical Informatics, Usher Institute (A.T.), University of Edinburgh, UK.; 3 Royal Infirmary of Edinburgh (C.C.), National Health Service Lothian, Edinburgh, UK.

**Keywords:** anxiety, psychotherapy, stroke, telemedicine, workflow

## Abstract

**Background and Purpose:**

Disabling anxiety affects a quarter of stroke survivors but access to treatment is poor. We developed a telemedicine model for delivering guided self-help cognitive behavioral therapy (CBT) for anxiety after stroke (TASK-CBT). We aimed to evaluate the feasibility of TASK-CBT in a randomized controlled trial workflow that enabled all trial procedures to be carried out remotely. In addition, we explored the feasibility of wrist-worn actigraphy sensor as a way of measuring objective outcomes in this clinical trial.

**Methods:**

We recruited adult community-based stroke patients (n=27) and randomly allocated them to TASK-CBT (n=14) or relaxation therapy (TASK-Relax), an active comparator (n=13).

**Results:**

In our sample (mean age 65 [±10]; 56% men; 63% stroke, 37% transient ischemic attacks), remote self-enrolment, electronic signature, intervention delivery, and automated follow-up were feasible. All participants completed all TASK-CBT sessions (14/14). Lower levels of anxiety were observed in TASK-CBT when compared with TASK-Relax at both weeks 6 and 20. Mean actigraphy sensor wearing-time was 33 days (±15).

**Conclusions:**

Our preliminary feasibility data from the current study support a larger definitive clinical trial and the use of wrist-worn actigraphy sensor in anxious stroke survivors.

**Registration:**

URL: https://www.clinicaltrials.gov. Unique identifier: NCT03439813.

## Abstract 2

Supplemental Digital Content is available in the text.

Anxiety affects around a quarter of stroke survivors and can be disabling even after minor stroke or transient ischemic attack (TIA)^1–3^ but psychological care is difficult to access.^4^ Randomized controlled trials (RCTs) have demonstrated that cognitive behavioral therapy (CBT) with guided self-help is effective for the treatment of anxiety in nonstroke populations.^5,6^ There is no definitive evidence to guide treatment for patients with stroke.^7,8^

Extra barriers to accessing face-to-face CBT for patients with stroke include physical immobility, the fear of going out alone, the fear of going to places full of people,^3^ and possible social stigma attached to seeking psychological treatments. Remotely delivered CBT by internet, email, telephone, mobile text messaging, or video calling (telemedicine) makes it possible to centralize resources for staff, training, quality monitoring, and cross coverage of large geographic areas, reducing cost and improving access. Meta-analyses have demonstrated guided internet-based CBT is superior to waitlist control and as efficacious as face-to-face CBT in treating anxiety disorders or depression in adults without stroke.^9,10^ We co-produced a telemedicine guided self-help CBT intervention with patients with anxiety after stroke (TASK-CBT).^11^ Additionally, we planned to use a wrist-worn actigraphy sensor (noninvasive method of monitoring rest and activity cycles) to obtain longitudinal data, offering potentially novel insights into daily living aspects of stroke survivors in community studies. Anxious stroke survivors showed higher levels of avoidant behavior across a range of situations, for example, going out alone, social situations, physical exertion compared with those who were not anxious.^3^ Sleep disturbances are clinical features in both anxiety and depression. Actigraphy offers the potential of gathering objectively measured data with minimal participant effort in clinical trials.

In this study, we aimed to demonstrate that it is feasible to (1) recruit and follow up remotely, (2) deliver CBT remotely, and (3) use an actigraphy sensor to collect continuous data in community-dwelling patients with stroke/TIA with anxiety.

## Methods

The data that support the findings of this study are available from the corresponding author on reasonable request as per the journal’s Transparency and Openness Promotion Guidelines.

The TASK RCT protocol was published^11^ and registered (20/2/2018) at ClinicalTrials.gov (NCT03439813). Favorable opinion was obtained from local research ethics committee (South East Scotland 17/SS/0143). All participants provided written informed consent using a web-form.

### Trial Design

**Figure 1.**
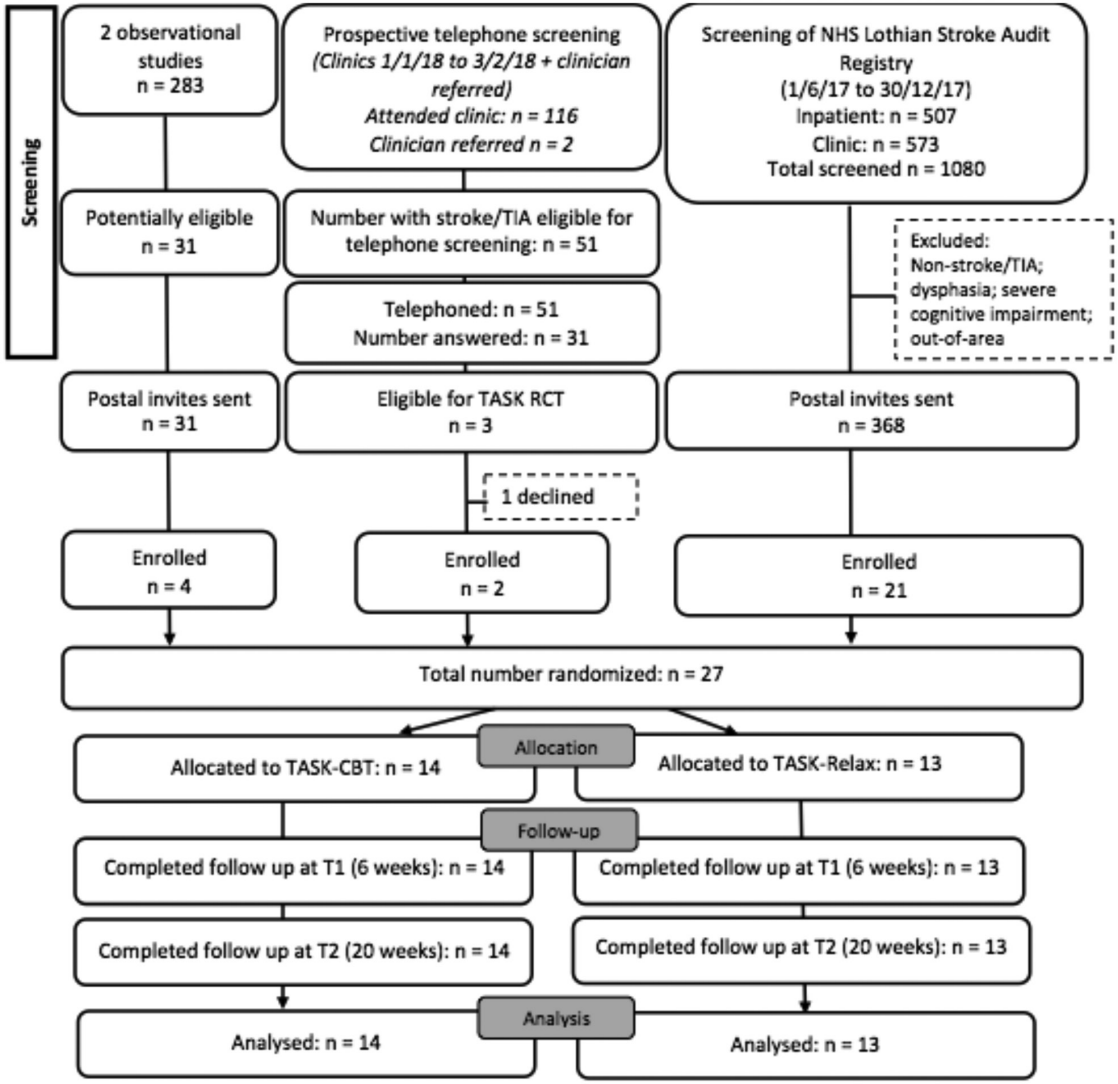
**Consort flow diagram. TIA indicates transient ischemic attack.**

The TASK feasibility trial was a parallel 2-armed RCT comparing TASK-CBT with relaxation control (TASK-Relax; Figure 1).

#### Patient Population—Inclusion and Exclusion Criteria

We recruited community-based adult stroke or TIA (probable, definite, or ocular) patients who were anxious (at least one positive response on our 6-item anxiety screening questions)^11^; had access to the internet and telephone; were able to speak English; gave informed consent; and lived within the catchment population of National Health Service Lothian—the sole provider of stroke and TIA services for a population of 800 000 people in the Lothian regions of Scotland, United Kingdom.

#### Recruitment Methods

Potential participants could view project information at www.task4stroke.org^12^ and complete informed consent with an electronic signature securely on this website, which uses the HIPAA-compliant Research Electronic Data Caputre (REDCap) application.^13^ We sent postal invitations to eligible participants identified from the National Health Service Lothian Stroke Audit register and previous observational studies. No financial incentives were offered. In addition, we offered trial information to stroke and TIA patients during their 1-month telephone follow-up.

#### Verifying Eligibility Remotely and Informed Consent

We verified eligibility with electronic health records. Baseline data were collected remotely including age, sex, stroke/TIA diagnosis, past history of, or medications for anxiety or mood; a telephone verbal fluency test (words beginning with F in one minute), modified Rankin Scale score,^14^ 7-item generalized anxiety disorder,^15^ modified fear questionnaire,^3,16^ patient health questionnaire-2,^17^ and EuroQol-5D5L.^18^

#### Intervention and Comparator

The TASK-CBT intervention was delivered remotely via the telephone, a treatment website, email, and mobile text. Participant allocated to TASK-CBT received 6 weekly telephone sessions (35–45 minutes each), delivered by the TASK therapist—a stroke physician (Dr Chun) trained in CBT with supervision from a consultant neuropsychiatrist (Dr Carson) using an electronic TASK therapist’s manual. Each week, the TASK-CBT participant received an online task to practise between sessions and was prescribed one or more of the psycho-educational videos on the TASK-CBT treatment website. Telephone sessions were audio-recorded for further validation of intervention fidelity.

Participants allocated to TASK-Relax had one introductory telephone session only. They were instructed to watch an introductory video and do 5 following online relaxation tasks: (1) audio and visually guided breathing exercise, (2) relaxing imagery and sounds, (3) music for relaxation, (4) audio-guided progressive muscle relaxation, and (5) a selection of sounds of nature. Participants selected a favorite task to practise daily for 5 minutes.

#### Components Common to Both Groups

To promote adherence and completion of follow-up surveys, participants of both arms received weekly mobile text reminders and a participant record card. All participants were aware that they would be offered access to the treatment website given to the other group after completion of their final follow-up survey at 20 weeks. Data collection was carried out at 6 and 20 weeks post-randomization using automated electronic self-completed surveys via emailed links.

#### Using a Wrist-Worn Actigraphy Sensor Throughout the TASK Trial

All participants were invited to wear the GENEactiv sensor,^19^ which records 3-dimensional acceleration (actigraphy), light, and wrist-temperature. Participants could wear the sensor on either wrist. After the first 2 months, the duration of the sensor’s rechargeable battery life, and with the participant’s agreement, a second sensor was sent to the participant to wear for the rest of the trial. All sensors were sent and retrieved by post.

#### Feasibility Outcomes

We assessed participants recruited per month, completion of electronic consent forms, time taken for remote eligibility confirmation (date of randomization—date of sign up and consent form received) and completion of electronic follow-up. We assessed adherence to TASK-CBT and intervention fidelity with (1) % completion of each online task, (2) aggregate web usage data from Google Analytics, and (3) review of audio-transcripts. We prespecified lack of feasibility as recruitment number of fewer than 2 participants per month; >50% of TASK-CBT patients dropping out after fewer than 3 telephone sessions; >10% noncompletion of follow-up surveys at 20 weeks, participants reporting unwanted effects from the intervention.

#### Clinical Outcomes

Treatment relevant outcomes included the modified Rankin Scale score, 7-item generalized anxiety disorder, modified fear questionnaire, patient health questionnaire-2, EuroQol-5D5L, and a single question about concurrent treatment for mood or anxiety. An online user feedback survey automatically followed the 20-week online follow-up survey. We assessed the feasibility of obtaining useable recording for at least 14 days for each sensor worn.

#### Sample Size

We aimed to recruit 40 participants in this feasibility RCT. We did not perform a statistical sample size calculation.

#### Randomization and Allocation Concealment

A researcher not involved in conducting the TASK trial generated a computerized permuted block randomization sequence with random block sizes. The sequence was uploaded to the in-built randomization module within the trial’s web-based data management application—REDCap^13^ and was inaccessible to the TASK researcher enrolling participants. On randomization, baseline data were collected remotely and participants were given their allocated treatment website. Participants allocated to TASK-CBT also received an appointment for their first telephone session.

#### Masking

The unblinded TASK researcher delivered the allocated intervention. We partially blinded participants by informing them they would be allocated to an anxiety treatment website. They were not aware of the contents of the intervention given to the other group.

### Statistical Methods

We used descriptive statistics to summarise patient characteristics, feasibility, and clinical outcomes. We used Google analytics^20^ for measuring aggregate web usage.

#### Mining the Actigraphy Data to Extract Characteristics

We used standard algorithms to mine the actigraphy data and computed activity and sleep characteristics^21–23^ at week 6 and week 20 post-randomization. We computed

1. Maximum average activity over 10 consecutive hours in a 24-hour day and is a measure of diurnal daily activity (M10),
2. Least average activity over 5 consecutive hours in a 24-hour day (L5),
3. Relative amplitude, takes into account M10 and L5: 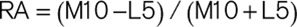
4. Inter-daily stability: expresses the stability of activity across days,
5. Intradaily variability: complementary to inter-daily stability, characterizing variability across days,
6. Mean diurnal activity during the 24 hours when the participant is not sleeping,
7. Mean nocturnal activity, mean activity during bedtime,
8. Percentile sleep activity, where we compute the 5th, 25th, 50th, 75th, and 95th activity percentile during bed time,
9. Sleep duration.

## Results

### Baseline Characteristics

We screened 1481 patients with a diagnosis of stroke or TIA and randomized 27 participants into TASK-CBT (n=14) and TASK-Relax (n=13) at a recruitment rate of 12 participants per month between January 27, 2018 and April 9, 2018 (Figure 1), finishing when funding came to an end. We recruited roughly equal numbers of men and women with a mean age of 65, who had experienced a minor stroke or TIA with mild disability (median modified Rankin Scale score=1). About a half had a history of anxiety or depression and had modest/severe anxiety at baseline (Table 1).

**Table 1.**
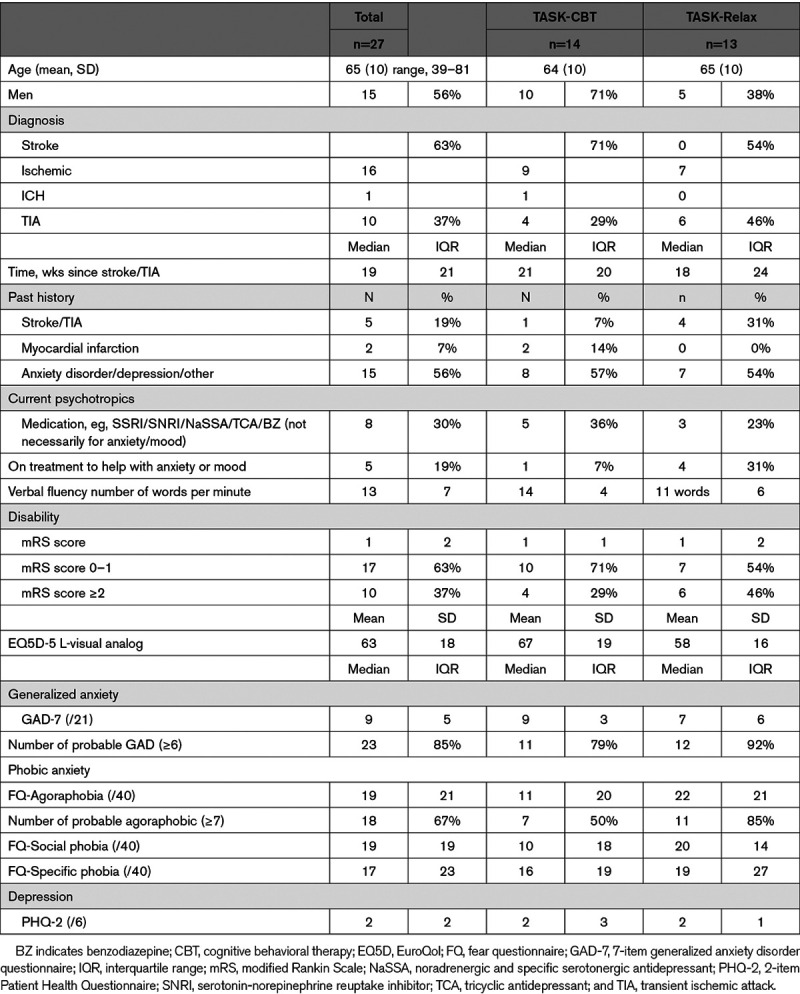
Baseline Characteristics (Prerandomization) of Sample

### Feasibility of Streamlined RCT Workflow Procedures

About a third of participants (10/27, 37%) completed an electronic consent form and the remainder by post. Confirming eligibility remotely took 1 day (IQR 3) from receiving consent to randomization. Hundred percent (27/27) completed follow ups at baseline, 6 weeks, and 20 weeks post-randomization.

### Feasibility of the TASK-CBT Intervention

All 14 participants allocated to the TASK-CBT group completed all 6 telephone sessions.

In the TASK-CBT group, online TASK completion was good for the first 3 online tasks but decreased over time (Table 2). Average time spent on the treatment website was similar in both groups (6 minutes per session) based on Google Analytics. Transcription of audio-recording was complete in only 51/84 TASK-CBT telephone sessions because of budget limits. We were not able to carry out independent reviews of audio-transcripts.

**Table 2.**
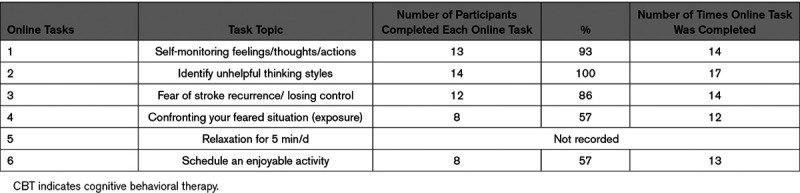
Completion of Online Tasks by TASK-CBT Participants (n=14)

### Clinical Outcomes

**Figure 2.**
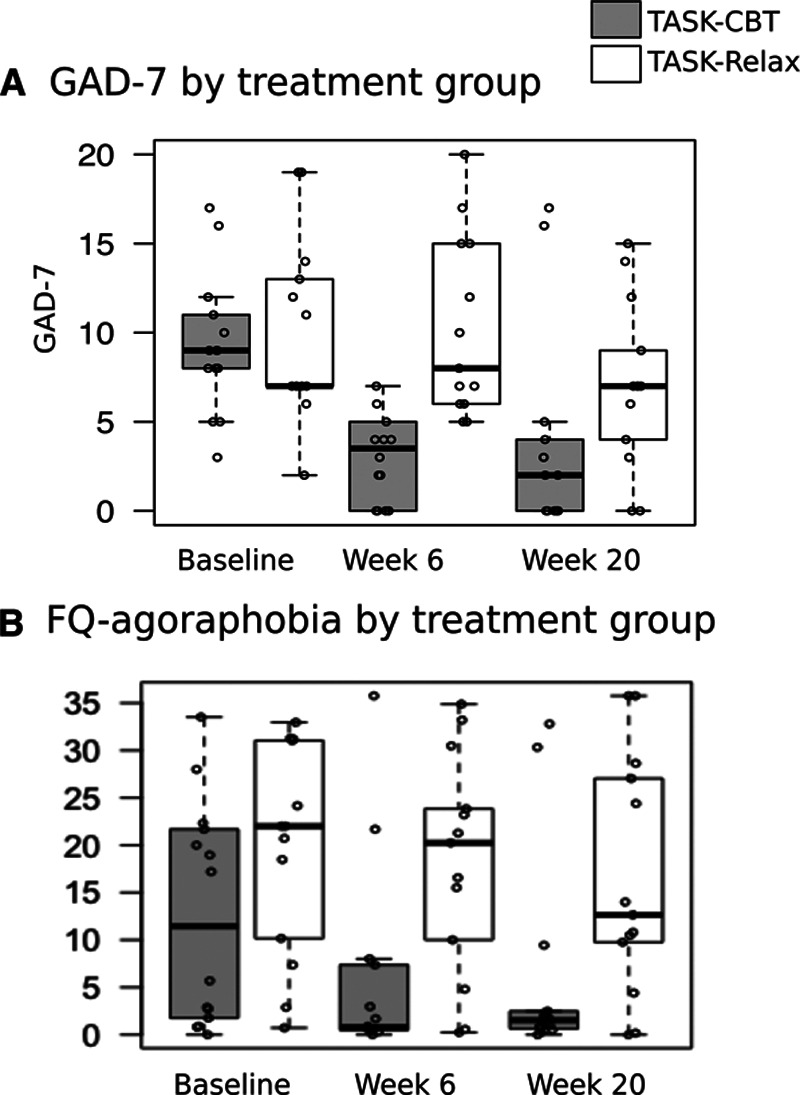
**Anxiety data by treatment group at week 6 and week 20 post-randomization.** Open dots represent data points. Box is interquartile range (IQR). Upper whisker extends from third quartile to the largest value no further than 1.5×IQR. Lower whiskers extends from first quartile to the smallest value at most 1.5×IQR. Data beyond the whiskers are outliers. FQ indicates fear questionnaire; and GAD-7, 7-item generalized anxiety disorder questionnaire.

Median modified Rankin Scale score was one in both groups at 6 and 20 weeks post-randomization. Lower levels of anxiety were observed at 6 and 20 weeks in the TASK-CBT group compared with the TASK-Relax group on the 7-item generalized anxiety disorder and FQ-agoraphobia (Figure 2). Median (IQR) score on GAD at 20 weeks was 2 (4) in TASK-CBT compared with 7 (5) in TASK-Relax. FQ-agoraphobic subscale at 20 weeks was 1.5 (1.9) in TASK-CBT compared with 12.6 (17) in TASK-Relax. Similar trends were found on FQ-social phobia and modified FQ-specific phobia (Supplement IA and IB in the Data Supplement).

### Participant Feedback

Participant feedback on their experience of using TASK-CBT was positive (Supplement II in the Data Supplement). Most participants allocated to TASK-CBT found the website easy to use. Participants reported accessing the treatment website at least 2 days a week. Most users reported finding the online videos and written information useful. Most participants reported the TASK-CBT treatment was helpful in overcoming their anxiety. No unwanted effects were reported. One participant felt slightly isolated.

### Feasibility Outcomes of Using Wrist-Worn Actigraphy Sensors

All 27 participants consented to wrist-worn actigraphy sensor for 2 months. 19/27 (74%) agreed to wear a second sensor. Useable recording was available for at least 14 days in 25/27 (93%) participants on the first sensor and 17/19 (89%) on the second sensor. Mean wearing time was 33 days (SD 15) on the first sensor and 35 days (SD 16) on the second (Supplement III in the Data Supplement). Reasons for declining to wear the sensor included wrist strap being too small, skin irritation, too bulky to wear, and already wearing another activity tracker. Data were not recorded in one sensor.

### Exploratory Actigraphy Data

**Figure 3.**
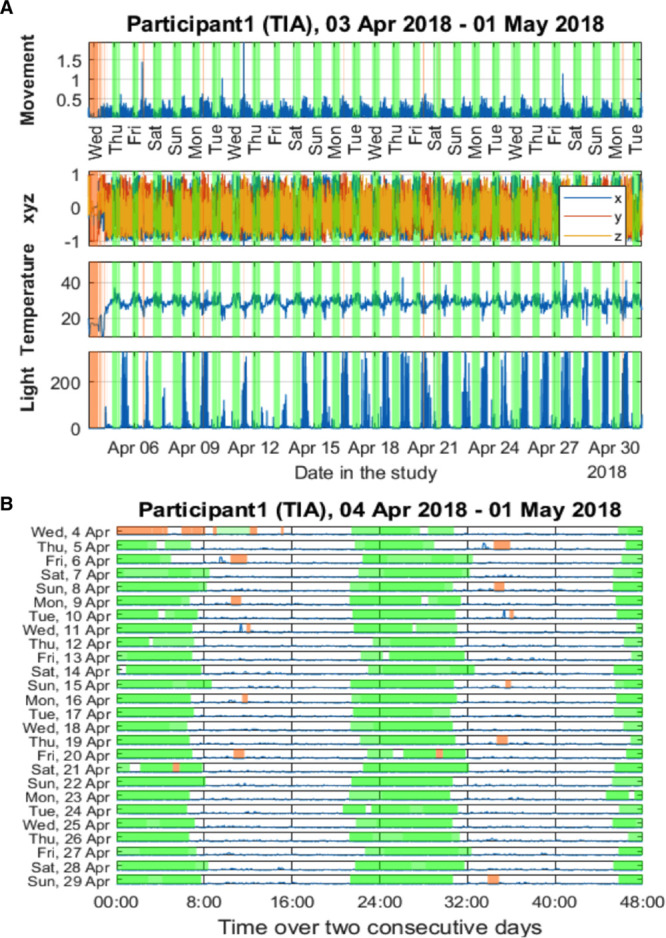
**Summary actigraphy data extracted from a GENEactiv sensor.** **A**, Summary of actigraphy data of a randomly selected participant (3-dimensional acceleration, wrist-temperature, and light) to illustrate data presentation. The vertical green transparent colour indicates automatically estimated sleep times. Transparent brown indicates nonwear times. **B**, Actogram plot for a randomly selected participant to illustrate data presentation. Green transparent colour indicates automatically estimated sleep times. Transparent brown indicates nonwear times. TIA indicates transient ischemic attack.

**Figure 4.**
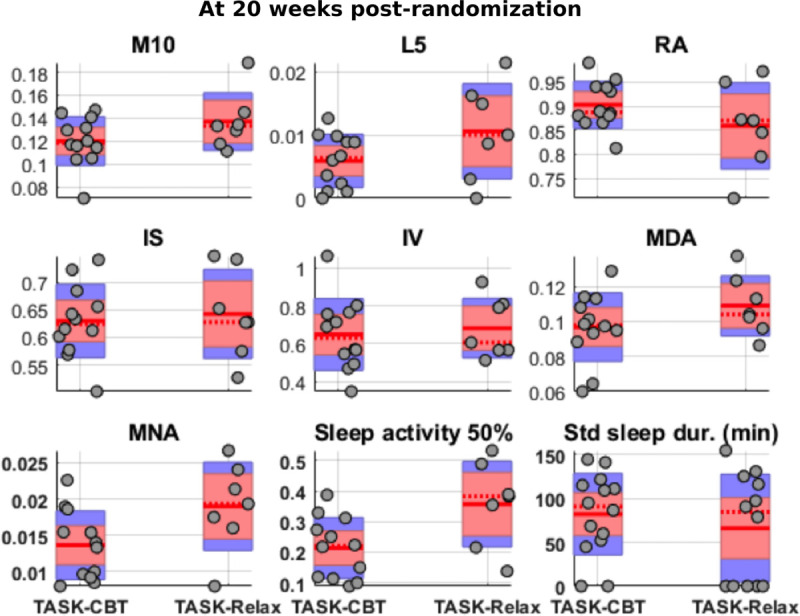
**Activity and sleep measures between TASK-CBT and TASK-Relax at 20 wk post-randomization.** IS indicates inter-daily stability; IV, intradaily variability; L5, least average activity over 5 consecutive hours in a 24-h day; M10, maximum average activity over 10 consecutive hours in a 24-h day; MDA, mean diurnal activity; MNA, mean nocturnal activity; 50th percentile sleep activity; sleep duration; and RA, relative amplitude.

We visualized the 3 data modalities (3-dimensional acceleration, wrist-temperature, light) for a randomly selected participant, presented as an actogram, a standard approach for visualizing actigraphy data in circadian biology (Figure 3). Figure 4 illustrates comparisons between 2 treatment groups (TASK-CBT versus TASK-Relax) across various objective actigraphy characteristics at 20 weeks (T2). There is some noticeable difference in sleep-related patterns using the mean nocturnal activity and median sleep activity (sleep activity 50%; Figure 4).

## Discussion

In this feasibility RCT of anxious stroke and TIA patients, we demonstrated it is feasible to conduct a clinical trial remotely for the delivery of telemedicine CBT for anxiety after stroke.

### Generalizability

Participants were relatively young, with mild disability, and could use the internet. Our sample size was small, and there were expected imbalances between the groups. Participants allocated to TASK-CBT took more antidepressants, were less disabled, and had less previous stroke/TIA than the TASK-Relax group, which could have biased the effect in favor of TASK-CBT. With wider adoption of home broadband by older people, populations who can use this intervention might increase.^24^ Telephone verbal fluency was included as an easy-to-administer screen of language and fluid-intelligence. Further assessment of expressive and receptive language and written communication should be considered in future studies.

### Limitations of Study Design

Screening the local stroke registry manually for eligible participants was time-consuming, thus limiting the number of patients contacted and generalizability of the study findings. Automation of the screening process and generation of study invitations could potentially overcome this challenge. Prospective recruitment was feasible but initial contact was opportunistic and made at the discretion of the stroke clinician during first presentation at clinic. A well-publicised online platform for use by stroke survivors to self-screen and self-recruit to research studies may help widen access to research studies. Lack of telephone sessions in TASK-Relax could have reduced adherence, which could be improved in a future trial with more telephone contact with these participants. Reviewing audio-recorded transcripts was not feasible given the time and funding constraints in this study. Google Analytics provided only aggregate but not individual web usage data so would not be a suitable tool as a measure of intervention fidelity. The automated data capture of online task completion by each participant offered a simple and feasible measure of intervention fidelity. We had a small sample size (14 in TASK-CBT and 13 in TASK-Relax) in this feasibility study with insufficient statistical power to draw conclusions on our clinical outcomes.

### Remote RCT Workflow

Our remote trial design could be applied to other remote-delivered interventions, for example, guided or unguided self-management. A remote trial design offers the potential to centralize trial staff and processes even in a large RCT. Screening with the local stroke registry was time-consuming, though verifying eligibility with electronic health records was feasible in one site and may be performed with national registers. Sending electronic proof of identity and eligibility to the research centre, for example, digital photograph, electronic clinic letter via a secure email, or form submission system are potential future options for national recruitment.

### Telemedicine Guided Self-Help CBT

TASK-CBT is designed as a low-intensity guided self-help CBT intervention delivered by appropriately trained and supervised stroke nurses or rehabilitation therapists. A telemedicine model could overcome the barriers in accessing psychological therapy by treating stroke patients remotely. We used relaxation as an active comparator, a therapy that may have benefit for generalized anxiety but has consistently been shown to be inferior to CBT-based intervention for panic, phobic, posttraumatic stress disorders in RCTs.^25^ Phobic anxiety, rather than generalized anxiety is the predominant subtype poststroke.^3^ Online relaxation therapy is popular, free, and widely used, making it a suitable comparator in a pragmatic RCT. RCTs of psychological therapy typically use treatment as usual as control condition which is known to exaggerate treatment effect.^26^

### Use of Wrist-Worn Sensors to Collect Continuous Actigraphy Data in an RCT

This study demonstrated the feasibility of using wrist-worn sensors in a stroke and TIA cohort and longitudinal use of the device to objectively assess therapy effects. A wrist-worn sensor is a convenient way to measure actigraphy data from which we can infer potentially clinically useful characteristics regarding a person’s activity and sleep patterns. These data could be clinically relevant in assessing anxiety after stroke. Passive collection of objective outcome measures throughout an entire RCT requires minimal participant effort, which in turn could minimize bias by reducing attrition, reliability, and inter-rater variability problems related to patient-reported outcome measures or observer-rated measures.

### Exploratory Data From an Actigraphy Sensor

We provided indicative plots for how data might be presented to visually assess patterns and have also extracted a number of known actigraphy measures to characterize long-term activity and sleep variability. We have also illustrated how the computed characteristics can be used to compare groups (TASK-CBT versus TASK-Relax in this study). We found that there is no obvious difference in terms of the extracted activity patterns during the daily activities, but there seems to be some noticeably different patterns in terms of the sleep characteristics computed using mean nocturnal activity and median sleep activity. We postulate the sleep patterns presented in Figure 4 might be reflecting anxiety effects, which would be in accordance with the findings on anxiety measures (Figure 2). The association of sleep and mental health (and in particular anxiety) is well reported in the research literature^27,28^; these findings tentatively support the argument that sleep characteristics might need to be further explored in association with CBT effects and anxiety management. This exploratory analysis paves the way for considering the use of wrist-worn actigraphy sensors in similar longitudinal studies both to assess inherent subject variability, potentially seasonality effects, therapeutic effects, and group cohort comparisons. Future work may investigate the association between objectively extracted information from actigraphy signals and patient-reported outcome measures or clinical assessments. Our small sample size limits further analyses of the actigraphy data.

## Conclusions

Telemedicine-delivered CBT is a feasible model to treat anxiety after stroke/TIA. A remote clinical trial workflow was feasible and will enable a definitive study. Wrist-worn actigraphy sensors could help collect objective outcomes in future stroke trials.

## Acknowledgments

We thank Surgical Informatics team at the Centre for Medical informatics, Usher Institute, University of Edinburgh, for their assistance with REDCap. All authors conceived the idea and contributed to the design of the current study. Dr Chun designed all study websites and the web-enabled trial procedures. She wrote all drafts and the final version of the manuscript with input from Drs Carson, Dennis, Tsanas, Mead, and Whiteley. Dr Calabria analyzed and wrote up the patient feedback data. Dr Tsanas developed the algorithms and conducted the analysis for the GENEActiv data. The study sponsor and funders had no role in the design or the reporting of the results of this study.

## Sources of Funding

Chief Scientist Office of Scotland Clinical Academic Fellowship (CAF/15/07) provided funding for the study and Dr Chun, a stroke physician (MBBS, MRCP, PhD). Lindsay Bequest and Reid Trust Grant funded some of the actigraphy sensors in this study (2017). Stroke Association’s Princess Margaret Research Development Fellowship supported HYC’s research training (2014–2015). Dr Whiteley is supported by the Chief Scientist Office of Scotland Senior Clinical Fellowship (CAF/17/01). The study was supported by Health Data Research UK, which receives funding from Health Data Research UK Ltd (HDR-5012) funded by the UK Medical Research Council, Engineering and Physical Sciences Research Council, Economic and Social Research Council, Department of Health and Social Care (England), Chief Scientist Office of the Scottish Government Health and Social Care Directorates, Health and Social Care Research and Development Division (Welsh Government), Public Health Agency (Northern Ireland), the British Heart Foundation, and the Wellcome Trust.

## Disclosures

Dr Carson is a paid associate editor of Journal of Neurology, Neurosurgery, and Psychiatry and is involved in 2 not-for-profit websites: www.neurosymptoms.org and www.headinjurysymptoms.org. He is a holder of a Health Technology Assessment grant in the United Kingdom to develop an app to deliver cognitive behavioral therapy after mild traumatic brain injury. This grant is shared with commercial partners. He has given independent testimony in Court on neuropsychiatric topics including anxiety after stroke. He has published and has been involved in a range of studies on the use of cognitive behavioral therapy for neurological diseases. He has received no personal payments from this but his research group benefits from high profile publications. Dr Mead reports royalties from Later Life Training and Elsevier outside the submitted work. She receives grants from the Stroke Association, Chief Scientist Office of Scotland, National Institute of Health Research, Chest Heart & Stroke Scotland outside the submitted work. She is a paid editor of Cochrane Stroke Group. Dr Whiteley reports grants from Chief Scientist Office of Scotland, Alzheimer’s Society and Stroke Association outside the submitted work.

## Supplementary Material


