# Supplementary material for: Telemedicine Cognitive Behavioral Therapy for Anxiety After Stroke: Proof-of-Concept Randomized Controlled Trial
Source: Stroke. 2020 Jul 24;51(8):2297–306. doi: 10.1161/STROKEAHA.120.029042 (PMC7382539; doi:10.1161/STROKEAHA.120.029042)
Supplement: Supplementary file 1 [file str-51-2297-s001.pdf]

Online Data Supplements

I. Between group differences on FQ-social phobia and FQ-specific phobia at 6 and 20 weeks. A) FQ-social phobia subscale by treatment group at 6 and 20 weeks post-randomization. FQ-social phobia score: TASK-CBT median 1.6, IQR 6.9; TASK-Relax median 10.5, IQR 24.2

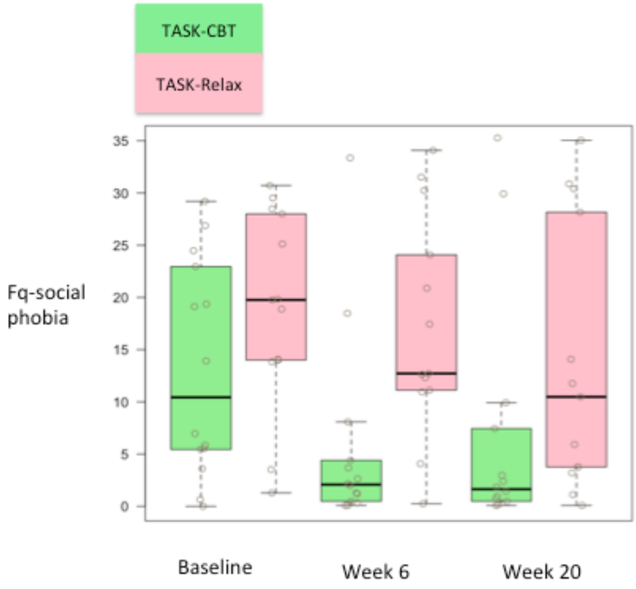

B) modified FQ-specific phobia subscale by treatment group at 6 and 20 weeks post-randomisation.

FQ-specific phobia score TASK-CBT median 2.12, IQR 6.6; TASK-Relax median 13.3 IQR 18.8

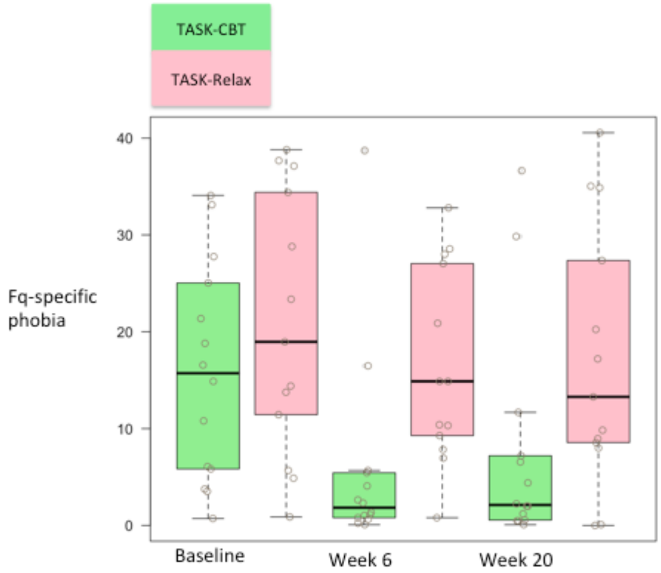

II. TASK-CBT participant free-text feedback

Positive feedback/ suggestions for improvement

- *Appreciated information on medication side-effects*
- *Increased flexibility during telephone consultations*
- *Patient felt they were 'overthinking the questions and getting lost'*

Technical issues using the TASK website

- *Difficulty selecting a specific number with sliders*
- *Suggestion regarding categorising videos by particular experience should more videos be added*

Weekly use of TASK-CBT website (n = 8)

**How often did you use your treatment website on average?**	
Less than 2 days a week (n=8)	1 (13%)
At least 2 days a week (n=8)	7 (88%)
Median number of days treatment website accessed	5.0

Treatment videos (n = 9)

**I found the online videos useful**	
Median	88
Number of patients who selected values ≤50	2 (22%)
Number of patients who selected values >50	7 (78%)

**Number of online videos watched**	
I watched 5 or more of the videos	6 (67%)
I watched 3 - 4 of the videos	2 (22%)
I watched 1 - 2 videos only	1 (11%)

Online written information (n = 9)

I found the online written information useful	
Median	92
Number of patients who selected a value ≤50	1 (11%)
Number of patients who selected a value >50	8 (89%)

Online tasks completed (n = 9)

Online tasks completed	
5 or more tasks completed	6 (67%)
2-4 tasks completed	3 (33%)
Only 1 task completed	0

Overcoming anxiety (n = 9)

Move slider to a position (0-100) to indicate how much TASK treatment has helped you overcome your anxiety (0: Not helped at all, 100: helped me overcome my anxiety completely)	
Median slider selection	77
Number of patients selecting a value of ≤50	1 (11%)
Number of patients selective a value of >50	8 (89%)

	I feel my TASK treatment has made my anxiety worse
Yes	0
No	9

Did you experience any unwanted effects from the TASK treatment?– free text (n=8)

- *It did make me think a bit more about my anxiety.*
- *None*
- *None whatsoever*
- *Slightly isolated*
- *Just keeping my attention during phone calls*
- *Not at all*
- *None at all. The online site and phone sessions were very positive.*
- *No*

III. Feasibility data on wrist-worn actigraphy sensor

	First watch	Second watch

No. Participants consented	27	19
No. with any useable recording	26	18
No. with useable recording > 7 days	25	17
No. with useable recording >14 days	22	16
No. with useable recording for 30 days	16	14
Mean wear time (days)	33 (SD 15)	35 (SD 16)
Issues with watch	Strap too small for 1 consenting participant. resulting in no data	No data available (device failure)

III Additional references

Additional references to the research literature demonstrating the use of the device GeneActriv and similar measures (M10, L5, RA and some other used here): https://www.activinsights.com/publications/
